# Long‐term trends in intergenerational proximity: Evidence from a grandchild design

**DOI:** 10.1002/psp.2473

**Published:** 2021-05-05

**Authors:** Matthijs Kalmijn

**Affiliations:** ^1^ Netherlands Interdisciplinary Demographic Institute‐KNAW University of Groningen The Hague Netherlands

**Keywords:** education, family, intergenerational geographic distance, rural–urban differences, urbanisation

## Abstract

Competing claims exist about how the geographic distance between parents and their adult children has changed historically. A classic modernisation hypothesis is that people currently live further away from their parents than in the past. Others have argued for stability and the remaining importance of local family ties, in spite of a long‐term decline in co‐residence of adult children and parents. The current paper uses a novel design that relies on reports by grandchildren to study long‐term changes in intergenerational proximity in the Netherlands. The analyses show that there has been a clear and continuous decline in intergenerational proximity between the 1940s and the 1990s. Mediation analyses show that educational expansion and urbanisation are the main reasons why proximity declined. No evidence is found for the role of secularisation and increasing international migration. Proximity to parents declined somewhat more strongly for women than for men.

## 1 INTRODUCTION

How close people live to their parents has been a topic of study in two fields. In family studies, the geographic distance between parents and children has been considered a dimension of family solidarity in an ageing society (Roberts et al., 1991). When adult children live close to their parents, they are more likely to have face‐to‐face contact with them and more likely to provide emotional and practical support to older parents who are in need (Hogerbrugge & Komter, 2012; Knijn & Liefbroer, 2006; Treas & Gubernskaya, 2012). The time and money it costs to travel play an obvious role in this association, but adult children who live close to their parents—and hence, have more regular contact—may also be more likely to recognise parents' support needs and hence provide more or more suitable care.

Geographic closeness to parents has also been a recurrent theme in studies of internal migration (Mulder, 2007). Migration not only depends on economic opportunities and household dynamics but also on the broader family context, that is, the dispersion of family members who do not live in the household. Considerations about the support that is needed from parents in taking care of grandchildren (Kulu & Milewski, 2007) as well as considerations about the health problems of older parents play a considerable role in decisions about moving or staying (Artamonova et al., 2020; Silverstein, 1995). In both cases, decisions about moving and staying depend at least in part on where other family members live. In the former stream of studies, intergenerational proximity is a determinant of support exchange between parents and adult children; in the latter stream of studies, intergenerational proximity is an outcome of (expected or desired) streams of support.

A long‐standing idea about intergenerational proximity is that adult children currently live further away from their parents than they did in the past (Litwak, 1960). Several aspects of the modernisation process supposedly contributed to such a trend, including educational expansion, upward occupational mobility, urbanisation, modern transportation and communication, and cultural processes such as individualisation and secularisation. Seemingly in contrast to this idea is the recurrent finding, also summarised in family textbooks (Cherlin, 2010, p. 328; Coltrane & Collins, 2001, p. 559), that most people nowadays live close to their parents. For example, in Europe, 75% of parents aged 50 and over without resident children have their nearest child living within 25 km (Hank, 2007). In the United States, 75% of the adults have a parent living within 30 mi. (Choi et al., 2020). There are different ways to quantify proximity and different designs, depending on whether one defines distance based on parent–child dyads or distance based on the nearest child (Choi et al., 2020). Nonetheless, current degrees of proximity do not suggest that there has been much historical change in proximity. Indeed, the consensus seems to be that local family ties have remained vital in postmodern society, except perhaps among the highest educated in society (Logan & Spitze, 1994).

There is little direct evidence on trends in intergenerational proximity. Four papers have systematically examined trends, but all of these analysed relatively short and recent periods. A study of Swedish register data showed that the distance between parents and adult children increased from 1980 to 2010, in line with the modernisation hypothesis (Chudnovskaya & Kolk, 2017). Similarly, in an analysis of the German Ageing Study, Steinbach et al. (2020) showed that the distance of parents to their adult children increased from 1996 to 2014. In contrast to these findings, a comparison of three British surveys conducted between 1986 and 1999 revealed stability in the proportion of daughters living within half an hour's journey time of their mother (Shelton & Grundy, 2000). A comparison of the 1986 and 2001 surveys from the International Social Survey Programme also showed stability in the mean travel time in all seven countries that were analysed (Treas & Gubernskaya, 2012).

Due to data limitations, it has been difficult to study trends for longer and older periods. Most national surveys on intergenerational relationships are relatively recent and therefore less suitable for studying long‐term trends. Census data are an important source of information on historical trends as well, but these have so far only been used for studying trends in the intergenerational co‐residence of adult children and parents (Ruggles, 2007). For an earlier period in history, the 19th century, there have been alternative types of data available. In a register study of two regions in Sweden, Hjälm (2011) showed a modest long‐term decline between 1820 and 1890 in the share of generations who lived in each other's vicinity, in combination with a strong decline in the share of generations who either co‐resided or lived extremely close to each other (Hjälm, 2011). In an analysis of 924 Dutch genealogies, Boele et al. (2018) compared the birth places of grandchildren and the place of death of grandparents, a plausible proxy for the geographic distance between adult children and their parents. Boele et al. (2018) showed that between 1,650 and 1850, there was a substantial decline in the share of grandchildren who were born in the same town or village where their grandparents died.

This paper used an indirect design to study trends in proximity in the 20th century over a longer period of time than previous studies were able to do. Specifically, I relied on a Dutch nationally representative survey in which respondents were asked questions about where their grandparents lived when they were growing up. The answers to these questions provided information on the intergenerational dyad formed by the parents and grandparents of the respondents. In other words, the design was to study respondents' parents in their role as adult children and respondent's grandparents in their role as parents. Because the respondents—that is, the grandchildren—were of all ages and because the questions pertained to their own youth, the data covered a long span of time. Specifically, the data allowed me to examine changes in parent–child proximity over nearly five decades, from 1943 to 1992.^1^ This was a period of rapid economic, cultural and demographic change, not only in the Netherlands but in most of the Western world (Inglehart, 1997).

Next to describing the trend, a second goal of this paper was to study factors that have been suggested as causes of the trend. Specifically, I analysed to what extent four societal processes affected the trend: educational expansion, urbanisation, secularisation and international migration.^2^ The first relevant process was educational expansion. As is well known, education is strongly negatively correlated with proximity: Higher educated children live further away from their parents than lower educated children (Chan & Ermisch, 2015a; Hank, 2007; Kalmijn, 2006; Michielin & Mulder, 2007). Several explanations have been given for this association. People who attend college often move out of their home community to study, the higher educated often rely on employment in urban areas and are generally more mobile in the labour market, and higher educated children and parents have family norms that are more individualistic. Educational expansion may be an important explanation for why proximity could have declined over time. One previous study found that a substantial portion of the decline in proximity of parents and adult children between 1980 and 2010 in Sweden was due to an increase in educational attainment (Chudnovskaya & Kolk, 2017).

A related process lies in educational homogamy, the tendency of people to marry within their educational group (De Hauw et al., 2017; Schwartz, 2013). In the current paper, the focus is on the intergenerational proximity of people who are married or cohabiting with a partner. For this stage in the life course, choices about where to live are made by two partners who each have to consider their own preferences, restrictions and their family network. As a result, the distance of a respondent to his or her parents will not only be affected by the respondent's education but also by the educational attainment of the partner. One would expect that in families with a traditional division of labour, the husband's education is more influential than the wife's education, but earlier studies did not find evidence of gender differences in the effects of education (Chan & Ermisch, 2015a). Educational homogamy—which has not declined over time in our study country, and even increased for the higher educated (Kalmijn & Uunk, 2016)—is thus expected to intensify the impact of educational expansion on a decline in proximity (Chudnovskaya & Kolk, 2017).

The second process was urbanisation. Past research has revealed substantial regional variations in intergenerational proximity, but the direction of this association depends on which generation one considers (Mulder & Kalmijn, 2006; van der Pers & Mulder, 2013). For adult children, there is a positive association between urbanisation and geographic distance, whereas for parents, there is a negative association. Children in urban areas live further away from their parents than children in rural (or less urban) areas, whereas parents in urban areas live closer to their children than parents in rural areas. The main reason for these associations lies in the process of rural–urban migration. If new generations of children mostly move from rural to urban areas, parents in rural areas are ‘left behind’ by children, whereas parents in urban areas are not. There also is return migration when adult children become parents themselves, sometimes to the rural areas where people grew up (Bijker & Haartsen, 2012; Kulu & Milewski, 2007; von Reichert et al., 2014). In the current paper, the focus is on the characteristics of the adult child generation, and the prediction is that urbanisation is positively related to geographic distance. Since the 1950s, there has been a trend towards more people living in urban areas although this trend slowed down over time and there have been signs of counter‐urbanisation (Fielding, 1989). For the period we study, we would expect that because an increasing number of people are living in urban areas, proximity to parents has declined over time.

Secularisation was a third trend that may have played a role in how intergenerational relations have changed over time. Religion is an important determinant of family solidarity. People who are religious tend to have stronger filial obligations than people who are not, and they also visit their parents more often and exchange more support with family members (Gans et al., 2009; Silverstein et al., 2019). As a result, one would expect that children and parents who are religious are more likely to live closer to each other than children and parents who are not religious. Similarly, families who are connected to more orthodox religious denominations would be more likely to live close to each other. Few studies have tested these associations, but one study in the United Kingdom found no association between having a religious affiliation and proximity to parents (Shelton & Grundy, 2000). If such an association would exist in the Netherlands, which will be examined, the decline in the share of people who are religious in the population may have contributed to a decline in proximity. Secularisation was closely related to both educational expansion and urbanisation (Halman & van Ingen, 2015), so the influences of religion, education and urbanisation must be studied in a multivariate fashion. Note that the increase in the share of people who identify as Muslims and Hindus will be a relevant countertrend (see below).

Finally, the increase in international migration may have played a role. In most European countries, the percentage of foreign‐born persons has increased steadily, in the Netherlands from 3.0% in 1947 to 10.6% in 2006 (CBS StatLine). The implications of this trend are not straightforward. On the one hand, one would expect that migration is positively associated with the geographic distance between parents and children because many immigrants have parents who are living in the country of origin (Chan & Ermisch, 2015b; Kalmijn, 2019; Valk & Bordone, 2019). On the other hand, there may be a negative association between migration and distance because (first‐ or second‐generation) immigrants who do have parents living in the destination country, more often live close to each other than natives. In a Dutch study, De Graaf et al. (2011) found that about a quarter of Turkish and Moroccan first and second‐generation immigrants aged 18–45 lived in the same neighbourhood as their parents, compared with only 10% among people aged 18–45 without a migration background, even after controlling for urbanisation, education and demographic characteristics (De Graaf et al., 2011).

Ideally, compositional changes in the number of immigrant children with and without parents in the destination country would be analysed, but this information is not available. An indirect approach would be to analyse first‐ and second‐generation immigrants because this is closely associated with having parents born abroad. Because of the grandchild design, the data do not allow me to define second‐generation immigrants as no information on the grandparents' country of birth was available. Although there has been an increase in the size of the second generation, the share of the second generation in the immigrant pool has not grown (from 52% in 1975 to 48% in 2000, CBS StatLine).

## 2 DATA, DESIGN AND METHOD

I used data from the first wave of the Netherlands Kinship Panel Study (NKPS) (Dykstra et al., 2004; Dykstra et al., 2005). The NKPS was based on a nationally representative sample of individuals in the Netherlands. Data for the first wave were collected in 2003, with small portions collected in 2002 and 2004. Of the initial sample, 37% was interviewed, 51% refused, and 12% could not be reached. The data are now almost two decades old, but for the current purpose, that was an advantage. The number of respondents was 8,161. I selected respondents in the ages of 18–65 (*N* = 7,051).

### 2.1 Design

**FIGURE 1:**
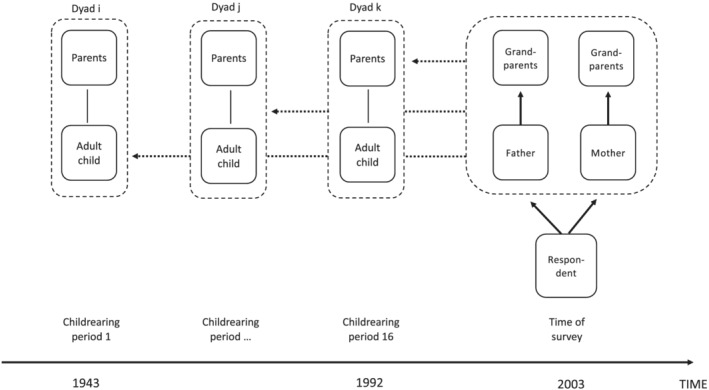
Design of the retrospective grandchild design

Respondents were asked where their two sets of grandparents lived when they were growing up. The parent–grandparent dyads constituted the units of analysis in this paper. For each respondent, there was information on two such dyads: between the mother and her parents and between the father and his parents. If a set of grandparents was no longer together, the grandparent who lived closest had to be selected.^3^ I excluded parent–grandparents dyads when the parent died before the respondent was 7 years of age (*n* = 128) and father–grandparent dyads when the parents divorced before the respondent was 7 years of age (*n* = 227).^4^ Dyads were also excluded if the respondent did not know where the grandparent lived (*n* = 27). The remaining number of dyads was 13,720. Dyads involving deceased grandparents were included in the descriptive part (Tables 1 and 2) but excluded in the regression analysis of proximity (Table 3; *n* = 12,447). In the remainder of this paper, the respondents' father and mother will be called *(adult) children*, and the respondents' grandparents will be called *parents*. The ‘grandchild design’ of the study is illustrated in Figure 1.

**TABLE 1:** Descriptive statistics

	Mean	SD	Min.	Max.	*N*
Parents deceased	.093		0	1	13,720
Parents in same place	.396		0	1	13,720
Parents in same place	.437		0	1	12,449
Adult child age	45.4	6.4	28	85	13,672
Child's education (ISLED)	4.22	2.19	1.66	9.46	12,923
Higher vocational education	.133		0	1	12,923
University education	.040		0	1	12,923
Child migrant	.079		0	1	13,253
Child level of urbanisation	2.89	1.32	1	5	12,972
Child Catholic	.444		0	1	11,738
Child Protestant (liberal)	.177		0	1	11,738
Child Protestant (orthodox)	.134		0	1	11,738
Child Islam/Hindu/other	.03		0	1	11,738

Source: NKPS Wave 1.

**TABLE 2:** Trends in the independent variables: regression estimates of time effects

Dependent variable	Time in decades		Constant	
	*b*	*t*	*b*	*t*
Adult child's age	−.967*	−21.6	45.379*	−842.6
Adult child education (ISLED)	.487*	−31.4	4.215*	−227.3
Higher vocational education (0/1)	.033*	13.4	.133*	44.7
University education (0/1)	.015*	10.1	.040*	23.2
Adult child migrant (0/1)	.017*	−8.5	.079*	−33.9
Very strongly urban (0/1)	.001	−0.4	.142*	−46.3
Strongly urban (0/1)	.017*	−5.5	.223*	−61.1
Moderately urban (0/1)	.019*	−6.5	.198*	−56.6
Hardly urban (0/1)	.007*	−2.1	.262*	−67.8
Not urban (0/1)	−.043*	−15.7	.176*	−53.1
Catholic (0/1)	−.019*	−5.0	.444*	−96.9
Liberal Protestant (0/1)	−.025*	−8.3	.177*	−50.3
Orthodox Protestant (0/1)	−.003	−1.0	.134*	−42.5
Non‐Western religion (0/1)	.012*	−8.8	.031*	−19.4

*Note*: Period is scaled in decades and mean centred. Source: NKPS Wave 1.

* *p* < .05.

**TABLE 3:** Linear probability model of adult child and parent living in the same town or city: coefficients and *t*‐values

	Model 1	Model 2	Model 3	Model 4	Model 5	Model 6
Period	−.043** (−8.10)	−.018** (−3.30)	−.035** (−6.54)	−.042** (−7.97)	−.043** (−8.12)	−.008 (−1.31)
Adult child's age	−.003** (−2.95)	−.003** (−2.75)	−.003** (−2.48)	−.004** (−2.94)	−.004** (−3.07)	−.003** (−2.43)
Daughter vs. son	−.060** (−6.20)	−.061** (−5.84)	−.060** (−6.22)	−.060** (−6.20)	−.062** (−6.44)	−.063** (−6.01)
Adult child education		−.028** (−9.71)				−.029** (−10.05)
Partner education		−.023** (−8.05)				−.024** (−8.56)
Very strongly urban^a^			−.183** (−8.63)			−.221** (−10.37)
Strongly urban^a^			−.160** (−8.10)			−.188** (−9.33)
Moderately urban^a^			−.167** (−8.08)			−.172** (−8.33)
Hardly urban^a^			−.093** (−4.71)			−.093** (−4.74)
Adult child migrant				−.052** (−2.01)		−.122** (−4.45)
Catholic					.033** (2.04)	.039** (2.43)
Liberal Protestant					.011 (.53)	.022 (1.14)
Orthodox Protestant					−.035** (−1.67)	−.015 (−.78)
Non‐Western religion					.059 (1.26)	.082 (1.63)
Constant	.735** (13.14)	.876** (16.36)	.805** (14.29)	.743** (12.81)	.731** (12.98)	.963** (17.19)
Mediation of time		−.024** (14.82)	−.008** (8.03)	.001** (0.98)	−.001** (1.90)	−.035** (16.15)
*N*	12,447	12,447	12,447	12,447	12,447	12,447

*Note*: Multiple imputation of missing values (no fit statistics). Source: NKPS Wave 1.

^a^ Rural is the reference category.

* *p* < .05.

** *p* < .10.

The main time variable used in the design was the period in which the adult children were raising the respondent. During the interview, childhood was defined as the time from birth to age 15. To define the historical observation period, I used the midpoint of the child‐rearing period (7 years). Respondents (grandchildren) were born between 1936 and 1985 with a median birth year of 1960. Using this variation, the period that I analysed began in 1943 and ended in 1992 (Figure 1). To facilitate the descriptive aims of the paper, the period variable was recoded into 16 3‐year intervals.^5^ In the regression models, the period variable was coded in years but rescaled in decades.

Before discussing the measures below, I discuss the challenges connected to the ‘grandchild’ design used in this paper. First, the trends applied to adult children who had children at home and not to adult children in general. As a result, the focus was on trends in proximity in one specific stage of the life course, that is, the child‐rearing stage. This was not necessarily a disadvantage because it is also a stage in which adult children often rely on support from their parents and a time when people have a desire to see their grandchildren (Chudnovskaya & Kolk, 2017; Geurts et al., 2009; Mulder, 2007). The transition to parenthood coincides with internal migration, with some counter‐migration occurring from cities to rural areas (Kulu & Milewski, 2007; von Reichert et al., 2014) and a decline in the distance between children and parents (Michielin & Mulder, 2007). Hence, the stage I considered was not representative of adult children in general but a stage in which adult children were more or less ‘settled’.

A second issue with the grandchild design for describing trends was that the sampling units were grandchildren. As a result, the sample was not representative of the population of interest because adult children who had a larger number of children were over‐represented in the data. To solve this problem as much as possible, I used weights based on the respondent's sibship size. Grandchildren with many siblings were given a lower weight than grandchildren with few siblings in the analysis. In doing so, the over‐representation of large families was corrected.

Third, the analyses may be affected by differential mortality. The survey included respondents (grandchildren) of all ages at the time of the survey, but especially at the higher ages, a large part of the birth cohorts will already have died. For example, a 90‐year‐old respondent could provide information on proximity about 80 years ago—an attractive piece of information from an historical perspective—but few 90‐year old people were still alive in 2004, the year when the survey was held. Because life expectancy was highly stratified by education (Mackenbach et al., 2008) and because intergenerational proximity varies by education (Kalmijn, 2006), this may bias the estimates of proximity for older periods. To reduce this bias, I excluded respondents who were older than 65 from the analysis. This choice seemed a reasonable trade‐off between obtaining as much historical breadth and as little mortality bias as possible. I will check how sensitive the results are for different age cut‐offs.

In the first part of the analysis, I described trends in intergenerational proximity. Second, I described changes in the variables that could mediate the trend: education, religion, urban residence and migration. Third, I presented analyses explaining the trend using a series of linear probability models. Models with and without additional independent variables were compared to assess to what extent these variables mediated the trend. In the last part of the analyses, I tested interaction effects with time. These analyses addressed the question of whether differences by education, urbanisation, religion and migrant status changed over time. Linear probability models were used given their straightforward interpretation and their flexibility for mediating effects (Mood, 2010). In all models, the standard errors were corrected for the clustering of dyads in families (male and female adult children came from couples).

### 2.2 Proximity

Intergenerational proximity was measured by assessing if adult children lived in the same town or city as their parents. There were four answering categories: (1) yes, during the entire (respondent's) childhood; (2) yes, during part of (respondent's) childhood; (3) no; or (4) (mother's/fathers's) parents no longer alive. The option ‘during part of childhood’ was uncommon (4.7%), pointing to the high degree of residential stability typically observed in the child‐rearing period. The variable was coded into a dichotomy, contrasting 1/2 to 3.^6^ When parents were no longer alive, the dyads were excluded from the regression analyses. In the descriptive part of the analyses, these dyads were included.

Because a retrospective design was used, the outcome variable was somewhat crude. No precise distance was measured, but the focus was on living in the same town or city. Although this variable had face validity and a good empirical distribution, it also had drawbacks. Living in the same town or city will mean something different in a city than in small town or rural area. Most cities in the Netherlands are small, however. The average population size of the 20 largest cities in the Netherlands was 254,000 in 2018, and the largest city (Amsterdam) had 854,000 inhabitants.^7^ Note further that towns have been reorganised legally and politically into larger municipalities. I am not aware of evidence suggesting that people identify with these larger units nor are there reasons to believe that respondents interpreted the survey question in terms of these legal/political units.

### 2.3 Independent variables

To statistically explain the trend, several independent variables were used. All measures applied to adult children and to the time they were raising their children. No information was available for the characteristics of the parents. Education was scaled in a linear fashion using the ISLED coding (Schröder & Ganzeboom, 2014) but was also treated as a categorical variable for comparisons (using six categories). Religion was measured by whether or not the child belonged to a church or religious denomination. This variable was dichotomised (0 = no, 1 = yes), but I also examined if there were differences between types of denominations by making a distinction between Catholics, liberal Protestants, orthodox Protestants and people with a non‐Western religious background (primarily Islam and Hindu). The degree of urbanisation was coded into five categories following procedures of Statistics Netherlands: very strongly urbanised (>2500 addresses/km^2^), strongly urbanised (1500–2500 addresses/km^2^), moderately urbanised (1000–1500 addresses/km^2^), hardly urbanised (500–1000 addresses/km^2^) and not urbanised or rural (<500 addresses/km^2^). Immigrant status was based on whether or not the child was born abroad. With the data, it was not possible to define the second generation. A possible second‐generation effect was partly covered by the non‐Western religious background variable.

The educational level of the adult child and his or her partner were used as parallel independent variables. Exploratory analyses were done to examine educational homogamy in detail using a distinction in three categories: husband higher educated than wife, wife higher educated than husband and husband and wife equal level of education (the reference category).^8^ These variables were included in a model that also contained a control for the couples' average level of education. Results for both men and women showed strong effects of the average level of education of the couple but insignificant effects of the two educational homogamy variables. For this reason, a specification using two main effects (of the adult child and the partner) was preferred.

Two control variables were used: the age of the adult child when his/her child was 7 years old. With this variable, I was able to adjust for possible changes in the timing of parenthood. I also controlled for the gender of the adult child. Previous studies examined if couples lived closer to the husband's or the wife's parents, but there was little consistent evidence in this direction (Chan & Ermisch, 2015a). Note that in the present set‐up, the gender effect applied to a comparison within couples (i.e., the respondents' father vs. mother). I tested interactions of gender and the other independent variables and will report when these were significant.

Missing values were imputed using multiple imputation with chained regression equations. Twenty imputations were done, and the results were combined using Rubin's rules (White et al., 2011). Means and standard deviations and the original *N*'s were presented in Table 1.

## 3 FINDINGS

### 3.1 Descriptive evidence

**FIGURE 2:**
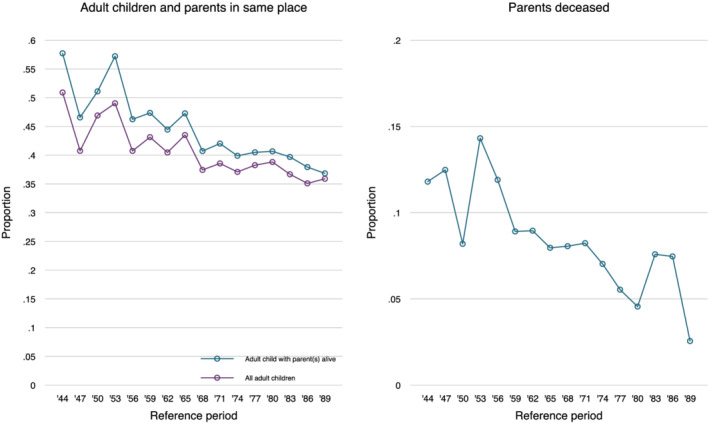
Proportion of adult children with deceased parents and with parents living in the same place by period

Figure 2 shows that the share of adult children who lived in same town or city as their parents declined over time. Among children who had at least one living parent when they were raising children themselves, the percentage living in the same place declined from about 50% to 55% in the 1940s and early 1950s to about 35% in the early 1990s (blue line). This supported the idea of a long‐term decline, but the magnitude of the change was not spectacular. Figure 2 shows that in this period, there also was a decline in the percentage of adult children whose parents were deceased. The main reason for this was most likely the increase in life expectancy. If we do not condition on having a living parent, the decline in proximity was somewhat smaller, as the red trend line in Figure 2 shows. In other words, the long‐term decline in proximity to parents was counteracted to some extent by increasing longevity of parents.

### 3.2 Changes in the compositional variables

Before turning to the explanatory analyses, I analysed how the independent variables changed over time (Table 2). The trend effects in Table 2 can be interpreted as the change in the dependent variable per decade. In line with expectations, there were strong trends for all key variables. The level of adult children's education increased, the proportion who were immigrants increased and the number of children who were church members declined. This decline was most apparent for Catholics and liberal Protestants. There was no change in the share of orthodox Protestants, and the share of people with a non‐Western religious affiliation increased. Urbanisation was also evident, with an increase in the share of adult children living in strongly and moderately urban areas and a corresponding decline in the share of adult children who lived in rural areas. There was no increase in the share of people living in the most urban areas. Note that the sample sizes varied due to missing values in the dependent variables.

### 3.3 Explaining the trend

Table 3 presents linear probability models explaining the probability that children lived in the same town or city as their parents. Model 1 only included the time variable. Subsequent models added sets of independent variables step by step. For each model, I assessed to what extent the effect of time was reduced when comparing a model with only the time variable and a model with the time variable and (sets of) independent variables. The change in the time effect across models, which was a measure of the statistical ‘explanation’ or ‘decomposition’ of the trend, was tested using a mediation module in Stata (Kohler & Karlson, 2012). The regression estimates were weighted for the inverse of the number of siblings. Table A1 presents unweighted estimates. Missing values were imputed so that the sample size was different than it was in Table 2.

Model 1 shows the effect of time and control variables. This trend effect was −.043 showing a 4 percentage‐point decline in proximity per decade. A test for non‐linearity showed that the quadratic effect was not significant (*p* = .734). The current sample was limited to respondents aged 18–65 to avoid possible bias due to (selective) mortality. I checked whether a more stringent age cut‐off would affect the trend parameter. Limiting the sample to ages 18–55 yielded the same trend (*b* = −.043). Broadening the sample, however, to ages 18–75 yielded a somewhat smaller trend parameter (*b* = −.033). This would be in line with selective mortality by education. In the oldest cohorts, the lower educated may have been under‐represented, leading to an underestimate of intergenerational proximity in these cohorts and hence a somewhat flatter trend line.

There were significant effects of the control variables. Older adult children were less likely to live close to their parents than younger adult children. Sons lived closer to their parents than daughters. Keep in mind that these findings applied to children who were raising children themselves.

Model 2 added educational variables. There were strong and significant effects of education. Higher educated children were less likely to live close to their parents and holding constant their own education, children with higher educated partners lived further away from their own parents compared with children with lower educated partners. The effect of time was reduced from −.043 to −.018 between Models 1 and 2, a reduction of 58%. At the bottom of the table, I tested if this reduction was significant using a mediation analysis. The test showed that the mediated effect was significant.

**FIGURE 3:**
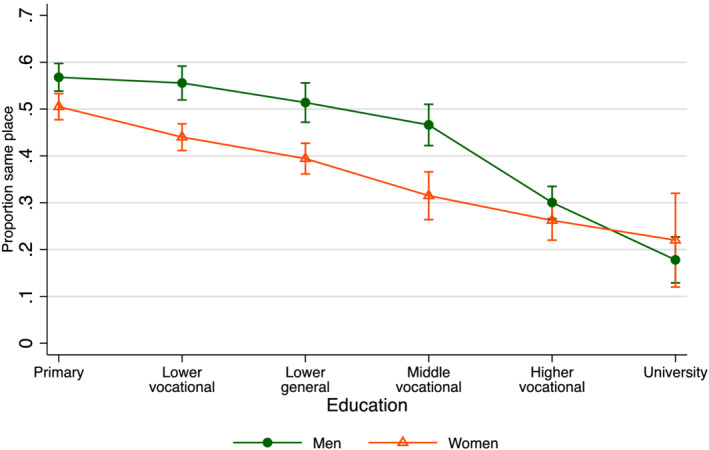
Margins of proximity by child's education and gender. Note: Margins obtained from a linear probability model with period, education, gender and the interaction of gender and education

To examine the effects of education in more detail, I plotted the effects in Figure 3 using a model with a categorical version of the education variable. The figure allowed us to see if the effects were linear and if the effects were similar for male and female adult children. Figure 3 shows that effects were indeed linear: Each higher level of education was associated with a smaller probability of living close to parents. The effects were also strong: Among lower educated men, nearly 60% lived in the same town or city as their parents; among university‐educated men, this was about 20%. The effects were somewhat stronger for men than for women, probably because of husbands' larger economic role in the families in these cohorts. An interaction of gender and education was significant (*b* = .014, *p* = .04). The interaction of gender and partner's education was not significant.

In Model 3, I added effects of urbanisation. The role of urbanisation was clear in Table 3. Adult children living in rural areas were the reference category. Each additional level of urbanisation was associated with a lower probability to live in the same place as the parents. Adult children in very strongly urban areas were least likely to live in the same place as the parents. The effect appeared more or less linear, although there was only a small difference between adult children in strongly and moderately urban areas. The effect of time declined to −.035 from Models 1 to 3, which amounts to a reduction of 19% of the trend. This reduction was significant.

Migration played a role as well, as Model 4 shows. Adult children who were foreign born were 5.2 percentage points less likely to live close their parents. The effect of time was hardly reduced when adding this variable. A possible explanation was that the share of immigrants was still rather small, which mitigated the impact on the overall trend.

Religion had small associations with proximity and in the expected direction (Model 5). Adult children who were Catholic were more likely to live close to their parents than secular children. An even larger effect was observed for persons who had a non‐Western religious denomination (5.9 percentage points), but this was not significant, probably due to the low number of Muslims and Hindus in the data. Interesting was that Protestants were not more likely to live close to their parents compared with secular persons; the religion effect was limited to Catholics and persons with a non‐Western religion. Because of these small effects and the opposing compositional changes (Table 2), there was no mediation of the trend.

In Model 6, all independent variables were included simultaneously. The effects of independent variables that were significant in the previous models remained significant in the full model, and some effects increased in size. Moreover, the effect of time was no longer significant. All the independent variables together explained 81% of the trend, a sizeable and significant portion. When looking at the separate models, it was clear, however, that education and to a lesser extent urbanisation were the prime components of this explanation.

**FIGURE 4:**
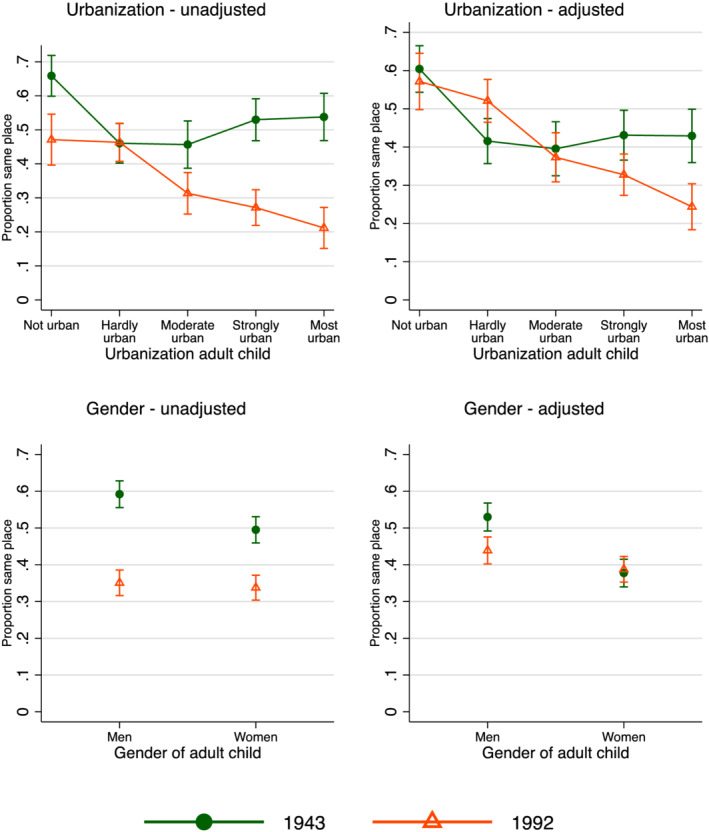
Margins of proximity by urbanisation, gender and period (margins obtained from Table 4)

In the last step of the analyses, I assessed if there were significant interactions of key independent variables with time (Table 4). Period interactions were assessed in two ways: with and without controlling for the (main) effects of the other covariates. The unadjusted results are in the left‐hand column; the adjusted models are in the right‐hand column. Margins for the main results are presented in Figure 4.

**TABLE 4:** Linear probability model of proximity with period interactions: coefficients and *t*‐values

	Unadjusted	Adjusted
Period	−.051* (−7.69)	−.016* (−2.38)
Daughter vs. son	−.099* (−4.56)	−.100* (−4.46)
x period	.015* (1.99)	.015** (1.90)
Period	−.039* (−3.03)	−.035* (−2.68)
Adult child education	−.034* (−5.09)	−.038* (−5.66)
x period	.002 (.97)	.003 (1.29)
Partner education	−.029* (−4.53)	−.033* (−5.09)
x period	.002 (1.08)	.003 (1.34)
Period	−.043* (−7.77)	−.007 (−1.15)
Adult child migrant	−.083 (−1.07)	−.056 (−.85)
x period	.011 (.47)	−.023 (−1.01)
Period	−.008 (−.63)	.010 (.81)
Adult child urbanisation	−.018** (−1.72)	−.038* (−3.62)
x period	−.011* (−2.80)	−.007** (−1.81)
Period	−.056* (−4.85)	−.020** (−1.75)
Adult child religious	−.035 (−.92)	−.022 (−.59)
x period	.019 (1.46)	.017 (1.35)
Non‐Western religion	.088 (.60)	.154 (1.11)
x period	−.010 (−.25)	−.027 (−.69)

*Note*: Multiple imputation of missing values (no fit statistics). Source: NKPS Wave 1.

* *p* < .05.

** *p* < .10.

There were no significant interactions of time with education, migration and religion. There was a significant interaction between time and gender, however. The unadjusted graph in Figure 4 shows that the decline in proximity was stronger for women than for men. The adjusted graph shows that the covariates explained a larger part of the trend for women than for men.

The interaction with urbanisation was also significant and in the negative direction, suggesting that urban–rural differences in proximity grew stronger over time. Figure 4 shows that there was no decline in proximity in the rural areas and in less urban areas, whereas there were stronger declines for children living in the most urban areas. Note that in the figure, urbanisation was treated as a non‐linear variable, whereas the test in Table 4 was based on a linear specification. Together, these differential trends led to a stronger effect of urbanisation in the more recent period.

**FIGURE 5:**
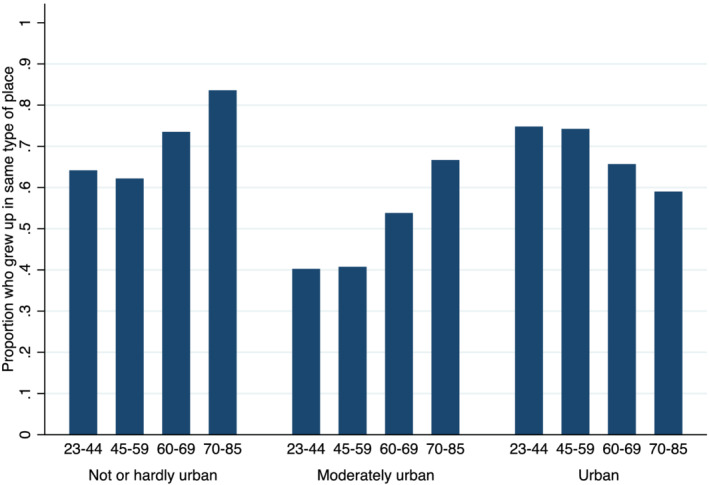
Proportion of adult children who lived in the same type of place as parents by current type of place of the adult child broken down by year of birth

To obtain more insight in the role of urbanisation, I compared the places where respondents lived at the time of the survey and the places where they lived when they were 15 years of age. For three levels of urbanisation (rural and hardly urban, moderately urban and very and strongly urban), I calculated the percentage of respondents who grew up in a similar type of place. Figure 5 presents the results separately for four birth cohorts. As expected, the share of persons in urban areas who also grew up in an urban area declined. Similarly, the share of persons in rural areas who also grew up in a rural area grew. In the most recent cohort, about 85% of the people in rural areas also grew up in a rural area, whereas only 59% of the urbanites grew up in an urban area. These changes were the result of increasing rural–urban migration and provided a possible explanation for why the association between urbanisation and intergenerational proximity increased over time: Recent urbanites were internal migrants more often. A caveat must be made that life course changes could not be considered in this extra analysis, so this may have biased the findings in Figure 5 to some extent.

## 4 CONCLUSION AND DISCUSSION

Using retrospective data and a novel design, this paper demonstrated a long‐term decline in proximity between parents and their adult children, in line with classic ideas in modernisation theory. The decline was clear but not spectacular in magnitude: About 50%–55% of the adult children lived in the same town or city as their parents after World War II; this declined to about 35% in the early 1990s. Proximity to parents declined for both genders but more strongly for women.

A large part of the trend was due to educational expansion, with a more modest role for urbanisation. There was no clear evidence that the role of education itself changed over time. The association between education and proximity to parents was strong and remained so over time. Because there was also a substantial expansion of education, this turned out to be the most important cause of the trend.

Secularisation did not contribute to the trend, in part because religious differences were small and inconsistent. The impact of secularisation was counteracted by a growth of the non‐Western religious population. Muslims, Hindus and persons with other non‐Western religions tend to live closer to their parents than secular persons. Migration also played a role, with first‐generation immigrants living further away from their parents, but the share of immigrants in the most recent period was too small to explain the trend.

My findings are consistent with an earlier Swedish study that analysed a shorter and more recent period (Chudnovskaya & Kolk, 2017). The data analysed in the current paper added a longer time window and suggest that trends in the 19th century, documented in regional register data in Sweden (Hjälm, 2011) and in Dutch genealogical data (Boele et al., 2018), continued in the 20th century.

My study also added a comparison of competing explanatory variables, which turned out to have effects on proximity, but effects not always large enough to explain the trend. My conclusion is in line with a more general idea in demography that structural change underlies family change (Blossfeld et al., 2005), and not so much cultural shifts like secularisation and individualisation (Lesthaeghe, 2014). Admittedly, cultural shifts were measured with only one simple—albeit important—variable. Still, education and urbanisation were also measured with one variable each, and these could explain a large part of the trend.

The role of education was clearly linear, suggesting that the location of universities was not the only reason for the association between education and intergenerational proximity. Labour market considerations will have played a role as well. Previous studies have demonstrated that education has a linear and positive causal effect on migration during the life course (Malamud & Wozniak, 2012). Other studies have shown that geographic mobility is an important way for the higher educated to avoid employment below one's educational qualifications (Hensen et al., 2009). In general, the educational effect on proximity will not solely depend on adult children moving away to attend university, although that will clearly be an element, but will also depend more generally on the geographic structure of the labour market for the better educated (Chan & Ermisch, 2015a).

Gender differences were observed as well. Husbands' education had a stronger effect on where couples lived than wives' education, a finding that is in line with an economic interpretation of the educational effect because in these cohorts, few mothers were employed and many mothers were employed part‐time (De Graaf & Vermeulen, 1997). Another important finding was that the decline in proximity was stronger for women than for men. This difference was probably due to the stronger rise in education for women (Buchmann et al., 2008) and the increase in married women's labour force participation in this period (De Graaf & Vermeulen, 1997).

The role of urbanisation requires more discussion. My analyses confirmed earlier studies by showing that people in urban areas live further away from their parents compared with people in rural areas (Michielin et al., 2008). This association stemmed largely from the role of rural–urban migration, a process that implied that people living in urban areas more often were internal migrants than people in rural areas. Moreover, I found that the trend in proximity was more pronounced for people in urban areas than for people in rural areas. People living in rural areas in the 1990s lived as close to their parents as people living in rural areas in the 1940s.

Although the grandchild design made it possible to look at long‐term changes, something that had not been possible with repeated cross‐sections in the past, the design also had limitations. First, the data only provided characteristics of adult children and not of parents. It is known where parents lived and if they were alive, but there was no other information on parents. It would have been ideal to analyse intergenerational proximity with characteristics from both sides of the dyad. Still, it is plausible that most of the ‘action’ will come from the adult child generation, as this is the more dynamic, more mobile member of the dyad. Previous studies confirmed that for proximity between adult children and parents, child characteristics were more influential than parent characteristics (Michielin & Mulder, 2007).

Second, the data can be affected by mortality. I used a cut‐off of age 65 to avoid this as much as possible, but even for this age range, more of the older respondents will already have died, and this will be more common for the lower educated than for the higher educated. This remains a disadvantage, but if this was a bias, it will probably work against my conclusions because the increase in educational attainment will have been underestimated rather than overestimated. Given these caveats, it remains important to also study changes in proximity using register data. Such data are available and important, but they are more limited in terms of their explanatory variables. In European countries where register data are available, information on education is often incomplete for older cohorts.

## CONFLICT OF INTEREST

The author declares that he has no conflict of interest.

## Data Availability

The data are available for public use from DANS‐KNAW (see https://dans.knaw.nl/en).

## ENDNOTES

1 A brief analyses of these data with a similar design was included as part of a descriptive paper on the NKPS by Mulder and Kalmijn (2006).

2 Declining family size was a potential explanation as well, but this could not be tested in this paper as there was no information on the number of siblings of the parents (i.e. the number of aunts and uncles), only on the number of parent's siblings who were alive at the time of the survey. This was so strongly affected by age that it could not be used.

3 The respondents' parents were born between 1868 and 1968 with a median of 1929. Historical research has shown that the experience of parental divorce in these cohorts was low. Of Dutch children born between 1923 and 1939, 3% had experienced the divorce of their parents before or at age 15 (van Poppel et al., 2013). For cohorts 1940–1964, the experience of parental divorce was still rare (4%). These numbers will be somewhat higher when the children were adults.

4 For these dyads, there was still information on proximity because the question was about the distance between the respondent (the grandchild) and the grandparent. The distance between grandchild and grandparent is not a good indicator of intergenerational co‐residence when parents were divorced or deceased.

5 To avoid using categories with very few cases, the first and last intervals contained 4 years (1943–1946 and 1989–1992).

6 Using a contrast of 1 to 2/3 yielded the same trend parameter.

7
https://opendata.cbs.nl/statline/#/CBS/nl/dataset/70748ned/table?ts=1606725858640

8 To simplify the homogamy analyses, education was coded in six ordered categories.

## APPENDIX A

**TABLE A1:** Unweighted linear probability model of adult child and parent living in the same town or city: coefficients and *t*‐values

	Model 1	Model 2	Model 3	Model 4	Model 5	Model 6
Period	−.041* (−9.61)	−.015* (−3.44)	−.034* (−7.90)	−.040* (−9.43)	−.040* (−9.48)	−.004 (−.85)
Adult child's age	−.002* (−3.00)	−.003* (−3.13)	−.002* (−2.44)	−.003* (−3.18)	−.003* (−3.23)	−.003* (−3.10)
Daughter vs. son	−.070* (−9.16)	−.076* (−9.02)	−.070* (−9.15)	−.071* (−9.17)	−.073* (−9.46)	−.078* (−9.27)
Adult child education		−.030* (−13.17)				−.032* (−13.64)
Partner education		−.023* (−9.68)				−.024* (−10.29)
Very strongly urban			−.154* (−8.68)			−.197* (−11.14)
Strongly urban			−.154* (−9.40)			−.187* (−11.42)
Moderately urban			−.172* (−10.10)			−.177* (−10.60)
Hardly urban			−.099* (−6.08)			−.100* (−6.31)
Adult child migrant				−.052* (−2.84)		−.112* (−5.18)
Catholic					.029* (2.18)	.036* (2.77)
Liberal Protestant					.018 (1.10)	.028** (1.74)
Orthodox Protestant					−.043* (−2.50)	−.026 (−1.56)
Non‐Western religion					.039 (1.26)	.049 (1.38)
Constant	.683* (16.37)	.850* (20.44)	.760* (17.96)	.693* (16.53)	.682* (16.06)	.954* (22.41)
*N*	12,449	12,449	12,449	12,449	12,449	12,449

*Note*: Multiple imputation of missing values (no fit statistics).

* *p* < .05.

** *p* < .10.

## References

[psp2473-bib-0001] Artamonova, A. , Gillespie, B. J. , & Branden, M. (2020). Geographic mobility among older people and their adult children: The role of parents' health issues and family ties. Population, Space and Place, 26(8), e2371. 10.1002/psp.2371 33935604 PMC8072412

[psp2473-bib-0002] Bijker, R. A. , & Haartsen, T. (2012). More than counter‐urbanisation: Migration to popular and less‐popular rural areas in the Netherlands. Population, Space and Place, 18(5), 643–657. 10.1002/psp.687

[psp2473-bib-0003] Blossfeld, H.‐P. , Klijzing, E. , Mills, M. , & Kurz, K. (Eds.) (2005). Globalization, uncertainty, and youth in society. London: Routledge.

[psp2473-bib-0004] Boele, A. , Störmer, C. , Gellatly, C. , & De Moor, T. (2018). Distant relatives? Demographic determinants of long‐term developments in intergenerational proximity, the Netherlands 1650–1899. The History of the Family, 23(3), 359–387. 10.1080/1081602X.2018.1454338

[psp2473-bib-0005] Buchmann, C. , DiPrete, T. A. , & McDaniel, A. (2008). Gender inequalities in education. Annual Review of Sociology, 34, 319–337. 10.1146/annurev.soc.34.040507.134719

[psp2473-bib-0006] Chan, T. W. , & Ermisch, J. (2015a). Proximity of couples to parents: Influences of gender, labor market, and family. Demography, 52(2), 379–399. 10.1007/s13524-015-0379-0 25794526

[psp2473-bib-0007] Chan, T. W. , & Ermisch, J. (2015b). Residential proximity of parents and their adult offspring in the United Kingdom, 2009‐10. Population Studies‐a Journal of Demography, 69(3), 355–372. 10.1080/00324728.2015.1107126 26585184

[psp2473-bib-0008] Cherlin, A. J. (2010). Public & private families: An introduction. McGraw‐Hill.

[psp2473-bib-0009] Choi, H. , Schoeni, R. F. , Wiemers, E. E. , Hotz, V. J. , & Seltzer, J. A. (2020). Spatial distance between parents and adult children in the United States. Journal of Marriage and Family, 82(2), 822–840. 10.1111/jomf.12606 33033415 PMC7537569

[psp2473-bib-0010] Chudnovskaya, M. , & Kolk, M. (2017). Educational expansion and intergenerational proximity in Sweden. Population, Space and Place, 23(1), 14. 10.1002/psp.1973

[psp2473-bib-0011] Coltrane, S. , & Collins, R. (2001). Sociology of marriage & the family. Wadsworth/Thomson Learning.

[psp2473-bib-0012] De Graaf, P. M. , & Vermeulen, H. (1997). Female labour‐market participation in the Netherlands: Developments in the relationship between family cycle and employment. In H.‐P. Blossfeld & C. Hakim (Eds.), Between equalization and marginalization: Women working part‐time in Europe and the United States of America (pp. 191–209). Oxford University Press.

[psp2473-bib-0019] De Graaf, P. M. , Kalmijn, M. , Kraaykamp, G. , & Monden, C. (2011). Sociaal‐culturele verschillen tussen Turken, Marokkanen en autochtonen: Eerste resultaten van de Nederlandse LevensLoop Studie (NELLS). Bevolkingstrends, 4, 61–70.

[psp2473-bib-0013] De Hauw, Y. , Grow, A. , & Van Bavel, J. (2017). The reversed gender gap in education and assortative mating in Europe. European Journal of Population, 33(4), 445–474. 10.1007/s10680-016-9407-z 30976234 PMC6241077

[psp2473-bib-0014] Dykstra, P. A. , Kalmijn, M. , Knijn, T. C. M. , Komter, A. E. , Liefbroer, A. C. , & Mulder, C. H. (2004). The Netherlands kinship panel study, 2002–2003. Dataset, available at DANS‐KNAW.

[psp2473-bib-0015] Dykstra, P. A. , Kalmijn, M. , Komter, A. E. , Liefbroer, A. , & Mulder, C. H. (2005). Codebook of the Netherlands kinship panel study, a multi‐actor, multi‐method panel study on solidarity in family relationships, wave 1. NKPS Working Paper No. 4. The Hague: Netherlands Interdisciplinary Demographic Institute.

[psp2473-bib-0016] Fielding, A. J. (1989). Counterurbanization in Europe: Migration and urbanization in Western‐Europe since 1950. Geographical Journal, 155, 60–69. 10.2307/635381

[psp2473-bib-0017] Gans, D. , Silverstein, M. , & Lowenstein, A. (2009). Do religious children care more and provide more Care for Older Parents? A study of filial norms and behaviors across five nations. Journal of Comparative Family Studies, 40(2), 187–201. 10.3138/jcfs.40.2.187 26203200 PMC4507809

[psp2473-bib-0018] Geurts, T. , Poortman, A. R. , van Tilburg, T. , & Dykstra, P. A. (2009). Contact between grandchildren and their grandparents in early adulthood. Journal of Family Issues, 30(12), 1698–1713. 10.1177/0192513x09336340

[psp2473-bib-0020] Halman, L. , & van Ingen, E. (2015). Secularization and changing moral views: European trends in church attendance and views on homosexuality, divorce, abortion, and euthanasia. European Sociological Review, 31(5), 616–627. 10.1093/esr/jcv064

[psp2473-bib-0021] Hank, K. (2007). Proximity and contacts between older parents and their children: A European comparison. Journal of Marriage and Family, 69(1), 157–173. 10.1111/j.1741-3737.2006.00351.x

[psp2473-bib-0022] Hensen, M. M. , de Vries, M. R. , & Corvers, F. (2009). The role of geographic mobility in reducing education‐job mismatches in the Netherlands. Papers in Regional Science, 88(3), 667–682. 10.1111/j.1435-5957.2008.00189.x

[psp2473-bib-0023] Hjälm, A. (2011). A family landscape: On the geographical distances between elderly parents and adult children in Sweden (dissertation). Umeå: Umeå University.

[psp2473-bib-0024] Hogerbrugge, M. J. A. , & Komter, A. E. (2012). Solidarity and ambivalence: Comparing two perspectives on intergenerational relations using longitudinal panel data. The Journals of Gerontology. Series B, Psychological Sciences and Social Sciences, 67(3), 372–383. 10.1093/geronb/gbr157 22357640

[psp2473-bib-0025] Inglehart, R. (1997). Modernization and postmodernization: Cultural, economic and political change in 43 societies. Princeton, N.J.: Princeton University Press.

[psp2473-bib-0026] Kalmijn, M. (2006). Educational inequality and family relationships: Influences on contact and proximity. European Sociological Review, 22(1), 1–16. 10.1093/esr/jci036

[psp2473-bib-0027] Kalmijn, M. (2019). Contact and conflict between adult children and their parents in immigrant families: Is integration problematic for family relationships? Journal of Ethnic and Migration Studies, 45(9), 1419–1438. 10.1080/1369183X.2018.1522245

[psp2473-bib-0028] Kalmijn, M. , & Uunk, W. (2016). Opleidingshomogamie in Nederland revisited: Stabiliteit of toenemende segmentatie op de huwelijksmarkt? In W. Jansen & I. Maas (Eds.), Scheidslijnen in Nederland (pp. 379–404). AUP.

[psp2473-bib-0029] Knijn, T. C. M. , & Liefbroer, A. C. (2006). More kin than kind: Instrumental support in families. In P. A. Dykstra , M. Kalmijn , T. Knijn , A. Komter , A. Liefbroer , & C. H. Mulder (Eds.), Family solidarity in the Netherlands (pp. 89–106). Dutch University Press.

[psp2473-bib-0030] Kohler, U. , & Karlson, K. (2012). KHB: Stata module to decompose total effects into direct and indirect via KHB‐method. Computer program.

[psp2473-bib-0031] Kulu, H. , & Milewski, N. (2007). Family change and migration in the life course: An introduction. Demographic Research, 17, 567–590. 10.4054/DemRes.2007.17.19

[psp2473-bib-0032] Lesthaeghe, R. (2014). The second demographic transition: A concise overview of its development. Proceedings of the National Academy of Sciences of the United States of America, 111(51), 18112–18115. 10.1073/pnas.1420441111 25453112 PMC4280616

[psp2473-bib-0033] Litwak, E. (1960). Geographic mobility and extended family cohesion. American Sociological Review, 25, 385–394. 10.2307/2092085

[psp2473-bib-0034] Logan, J. R. , & Spitze, G. (1994). Family neighbors. American Journal of Sociology, 100, 453–476. 10.1086/230543

[psp2473-bib-0035] Mackenbach, J. P. , Stirbu, I. , Roskam, A. J. R. , Schaap, M. M. , Menvielle, G. , Leinsalu, M. , Kunst, A. E. , & European Union Working Grp, S . (2008). Socioeconomic inequalities in health in 22 European countries. New England Journal of Medicine, 358(23), 2468–2481. 10.1056/NEJMsa0707519 18525043

[psp2473-bib-0036] Malamud, O. , & Wozniak, A. (2012). The impact of college on migration evidence from the Vietnam generation. Journal of Human Resources, 47(4), 913–950.

[psp2473-bib-0037] Michielin, F. , & Mulder, C. H. (2007). Geographical distances between adult children and their parents in the Netherlands. Demographic Research, 17, 655–677. 10.4054/DemRes.2007.17.22

[psp2473-bib-0038] Michielin, F. , Mulder, C. H. , & Zorlu, A. (2008). Distance to parents and geographical mobility. Population, Space and Place, 14(4), 327–345. 10.1002/psp.509

[psp2473-bib-0039] Mood, C. (2010). Logistic regression: Why we cannot do what we think we can do, and what we can do about it. European Sociological Review, 26(1), 67–82. 10.1093/esr/jcp006

[psp2473-bib-0040] Mulder, C. H. (2007). The family context and residential choice: A challenge for new research. Population, Space and Place, 13(4), 265–278. 10.1002/psp.456

[psp2473-bib-0041] Mulder, C. H. , & Kalmijn, M. (2006). Geographical distances between family members. In P. A. Dykstra , M. Kalmijn , T. C. M. Knijn , A. E. Komter , A. C. Liefbroer , & C. H. Mulder (Eds.), Family solidarity in the Netherlands (pp. 63–88). Dutch University Press.

[psp2473-bib-0042] Roberts, E. L. , Richards, L. N. , & Bengtson, V. L. (1991). Intergenerational solidarity in families: Untangling the ties that bind. Marriage & Family Review, 16(1–2), 11–46. 10.1300/J002v16n01_02

[psp2473-bib-0043] Ruggles, S. (2007). The decline in intergenerational coresidence in the United States, 1850‐2000. American Sociological Review, 72, 964–989. 10.1177/000312240707200606 21562613 PMC3090139

[psp2473-bib-0044] Schröder, H. , & Ganzeboom, H. B. G. (2014). Measuring and modelling level of education in European societies. European Sociological Review, 30(1), 119–136. 10.1093/esr/jct026

[psp2473-bib-0045] Schwartz, C. R. (2013). Trends and variation in assortative mating: Causes and consequences. Annual Review of Sociology, 39(39), 451–470. 10.1146/annurev-soc-071312-145544

[psp2473-bib-0046] Shelton, N. , & Grundy, E. (2000). Proximity of adult children to their parents in Great Britain. International Journal of Population Geography, 6, 181–195. 10.1002/1099-1220(200005/06)6:3<181::AID-IJPG181>3.0.CO;2-U 12349713

[psp2473-bib-0047] Silverstein, M. (1995). Stability and change in temporal distance between the elderly and their children. Demography, 32(1), 29–46. 10.2307/2061895 7774729

[psp2473-bib-0048] Silverstein, M. , Zuo, D. M. , Wang, J. P. , & Bengtson, V. L. (2019). Intergenerational religious participation in adolescence and provision of assistance to older mothers. Journal of Marriage and Family, 81(5), 1206–1220. 10.1111/jomf.12592 32863428 PMC7451961

[psp2473-bib-0049] Steinbach, A. , Mahne, K. , Klaus, D. , & Hank, K. (2020). Stability and change in intergenerational family relations across two decades: Findings from the German ageing survey, 1996‐2014. The Journals of Gerontology. Series B, Psychological Sciences and Social Sciences, 75(4), 899–906. 10.1093/geronb/gbz027 30901059

[psp2473-bib-0050] Treas, J. , & Gubernskaya, Z. (2012). Farewell to moms? Maternal contact for seven countries in 1986 and 2001. Journal of Marriage and Family, 74(2), 297–311. 10.1111/j.1741-3737.2012.00956.x

[psp2473-bib-0051] Valk, H. A. G. , & Bordone, V. (2019). Co‐residence of adult children with their parents: Differences by migration background explored and explained. Journal of Ethnic and Migration Studies, 45(10), 1790–1812. 10.1080/1369183x.2018.1485207

[psp2473-bib-0052] van der Pers, M. , & Mulder, C. H. (2013). The regional dimension of intergenerational proximity in the Netherlands. Population, Space and Place, 19(5), 505–521. 10.1002/psp.1729

[psp2473-bib-0053] van Poppel, F. , Schenk, N. , & van Gaalen, R. (2013). Demographic transitions and changes in the living arrangements of children: The Netherlands 1850‐2010. Population Research and Policy Review, 32(2), 243–260. 10.1007/s11113-012-9264-3

[psp2473-bib-0054] von Reichert, C. , Cromartie, J. B. , & Arthun, R. O. (2014). Reasons for returning and not returning to rural US communities. The Professional Geographer, 66(1), 58–72. 10.1080/00330124.2012.725373

[psp2473-bib-0055] White, I. R. , Royston, P. , & Wood, A. M. (2011). Multiple imputation using chained equations: Issues and guidance for practice. Statistics in Medicine, 30, 377–399. 10.1002/sim.4067 21225900

